# Comparative Evaluation of Root Canal Transportation by Three NiTi Single-File Systems in Curved Canals: A Cone Beam Computed Tomography Study

**DOI:** 10.1155/2018/4151692

**Published:** 2018-05-14

**Authors:** Eshaghali Saberi, Narges Farhad-Mollashahi, Shima Bijari, Mohammad Daryaeian

**Affiliations:** Department of Endodontics, Oral and Dental Disease Research Center, Dental School, Zahedan University of Medical Sciences, Zahedan, Iran

## Abstract

**Introduction:**

This study is aimed at evaluating root canal transportation in the mesiobuccal canal of mandibular first molars prepared with One Shape, Reciproc, and M-One nickel titanium (NiTi) single-file rotary systems using cone beam computed tomography (CBCT).

**Materials and Methods:**

In this ex vivo study, CBCT scans of 45 extracted human mandibular first molars with 20–40° curvature were obtained. The teeth were randomly divided into three groups (*n*=15) for preparation of the mesiobuccal canal with One Shape, Reciproc, and M-One rotary systems according to the manufacturers' instructions. CBCT scans were obtained again after canal preparation. Changes caused by preparation in the coronal, middle, and apical thirds were determined on CBCT scans and analyzed using the Kruskal–Wallis test at *P* ≤ 0.05 level of significance.

**Results:**

No significant difference was noted in the amount of canal transportation among the three groups (*P* > 0.05). M-One caused greater transportation in the apical third compared with Reciproc and One Shape, and One Shape caused greater transportation in the coronal third compared with other groups, although its magnitude was less than 0.3 mm.

**Conclusion:**

Reciproc, One Shape, and M-One are not significantly different in terms of canal transportation.

## 1. Introduction

The main objective of endodontic treatment is to eliminate or minimize microorganisms in the root canal system while maintaining the original shape and path of the root canal [1]. However, no instrument/technique can predictably eliminate all the microorganisms from the root canal system. The cleaning efficacy of endodontic instruments significantly decreases at the apical third of root canals [2]. Many root canals have curvatures, and endodontic instruments tend to return to their original straight position during instrumentation of curved canals [3, 4]. Dentinal wall thickness in root canals has a direct relationship with root resistance to lateral forces [5, 6].

In the recent years, several nickel titanium (NiTi) instruments capable of faster and more efficient root canal preparation were introduced in the market. These systems have differences in some features such as cleaning efficacy, stress applied to dentinal walls, and ability to prepare oval-shaped root canals [7].

One Shape file (MicroMega, France) operates with continuous rotational movement compared with the other single-file systems. One Shape instruments have higher cutting efficacy in the root, which is probably attributed to electropolishing, flexibility, and variable cross section along its blade [8]. Antibreakage control in this file increases its fracture strength. This system has a sterile file with a tip size of ISO 25, 0.06 taper, variable pitch and a noncutting (safety) tip for cleaning and shaping of root canals [5].

Reciproc file (VDW, Munich, Germany) is made of M-Wire, which increases its strength and flexibility [9]. This file has an S-shaped cross section and a noncutting (safety) tip operating with reciprocal motion. Reciprocal movement was introduced in 1985 and is composed of two rotations, namely, 150° counterclockwise and 30° clockwise motions. This file is available in three sizes and tapers: R25 (25/0.08), R40 (40/0.06), and R50 (50/0.05) [10, 11].

M-One (Park, China) is a single-file system with a tip size of ISO 25, 0.06 taper, and full rotational motion. The alloys used in its composition are CM-Wire and Neo NiTi, and it has a regular triangular cross section.

Cone beam computed tomography (CBCT) can be used to assess the amount of dentin removed from the root canal walls during root canal preparation. This imaging modality enables assessment of volume, surface area, cross-sectional shape, and taper of canal with no damage to tooth structure [8].

A previous study showed that WaveOne with reciprocating motion caused less canal transport than One Shape and ProTaper files [12]. Another study showed that Reciproc and WaveOne instruments caused significantly less canal transport than One Shape [13], while Cimilli and Kartal [14] indicated that continuous rotation had higher centering ability than reciprocating motion.

No previous study has compared canal transportation caused by three NiTi single-file systems, namely, One Shape and M-One with continuous rotational movement and Reciproc with reciprocal movement. Thus, this study aimed at comparing canal transportation in the mesiobuccal canal of mandibular first molars prepared with One Shape, Reciproc, and M-One NiTi single-file rotary systems using CBCT.

## 2. Materials and Methods

The study was approved by the Ethics Committee of Zahedan University of Medical Sciences (IR.ZAUMS.REC.1395.83), and written informed consent was obtained from all patients. The study was conducted on 45 extracted mandibular first molars of patients presented to the Oral and Maxillofacial Surgery Department of Zahedan University of Medical Sciences. The teeth had been extracted due to periodontal or orthodontic reasons and had closed apices and mesial root curvature of 20–40° measured according to Schneider's method [15]. Root curvature measured 5–9 mm distance from the apex, and the mean length of the root was 19–22 mm.

After collection of teeth, tissue residues and calcified debris were eliminated, and the teeth were disinfected in 0.1% thymol solution at 9°C for 24 hours. The teeth were rinsed with tap water to eliminate thymol residues, and they were then immersed in saline at 4°C. Primary radiographs of the mesial root were obtained to determine the degree of root curvature. Teeth with mesial root canals with one apical foramen and no sign of calcification or internal resorption were included in the study. Teeth with S- or C-shaped canals were excluded. All roots were evaluated under a stereomicroscope at ×12 magnification to ensure absence of craze lines, cracks, or fractures. Teeth with such defects were excluded from the study and replaced with sound teeth. Three-dimensional CBCT scans were obtained with the Vatech 3D system (Ez3D Plus, Korea) with the exposure settings of 89 kVp, 5.4 mA, 50 × 50 mm field of view, 0.08 mm voxel size, and 10 s time prior to preparation of root canals. Access cavity was prepared by a diamond bur and high-speed handpiece under air and water spray to negotiate mesiobuccal canal orifice. To determine the mesiobuccal canal working length, a #10 K file (Mani, Tochigi, Japan) was introduced into the canal until its tip was visible at the apex. The working length was determined to be 1 mm shorter than this length.

A silicone impression material (Oranwash; Zhermack spa, Rovigo, Italy) was used to cover the cementum to simulate periodontal ligament. To prevent the entry of the silicone material into the apical foramen, the apex was sealed with red dental wax. The teeth were then mounted in blocks measuring 5 × 5 mm filled with putty wash to the level of cementoenamel junction. The teeth were embedded in a mold in a parallel fashion to standardize the pre- and postinstrumentation images. A small piece of orthodontic wire was placed at the corner of silicone blocks as a guide to ensure correct direction of scanning.

The teeth were randomly divided into three groups of 15. Reciproc rotary file was used in group 1, One Shape was used in group 2, and M-One was used in group 3.

### 2.1. Root Canal Preparation

All mesiobuccal canals were instrumented to the working length using the crown-down technique with a handpiece (X-Smart, Dentsply Maillefer, Japan) at the speed and torque recommended by the manufacturers for each system. The root canals were irrigated with 2.5% sodium hypochlorite solution delivered with a 30-gauge needle between instruments. Also, 17% EDTA and 5.25% sodium hypochlorite were used for final rinse and elimination of smear layer.

### 2.2. Root Canal Preparation in Group 1

Reciproc file with a tip size of ISO 25 and 0.08 taper was used to reach the working length with gentle pecking motion and reciprocating rotation at the speed and torque recommended by the manufacturer. Recapitulation was done frequently using a #10 K file, and the mesiobuccal canals were rinsed with 2.5% sodium hypochlorite after using each instrument. Glyde was used as the lubricant.

### 2.3. Root Canal Preparation in Group 2

One Shape file with a tip size of ISO 25 and 0.06 taper was used with continuous rotation and gentle in-and-out movement at the speed and torque recommended by the manufacturer to reach the working length. Recapitulation was done repeatedly using a #10 K file. The mesiobuccal canals were rinsed with 2.5% sodium hypochlorite after using each instrument. Glyde was used as the lubricant.

### 2.4. Root Canal Preparation in Group 3

M-One file with a tip size of ISO 25 and 0.06 taper was used to reach the working length with continuous rotation and torque recommended by the manufacturer. Recapitulation was done repeatedly using a #10 K file. The mesiobuccal canals were rinsed with 2.5% sodium hypochlorite after using each instrument. Glyde was used as the lubricant.

CBCT scans of the teeth were obtained with the same settings mentioned earlier. The root canal wall thickness in uninstrumented and instrumented root canals was measured at 3, 6, and 9 mm from the apex.

**Figure 1:**
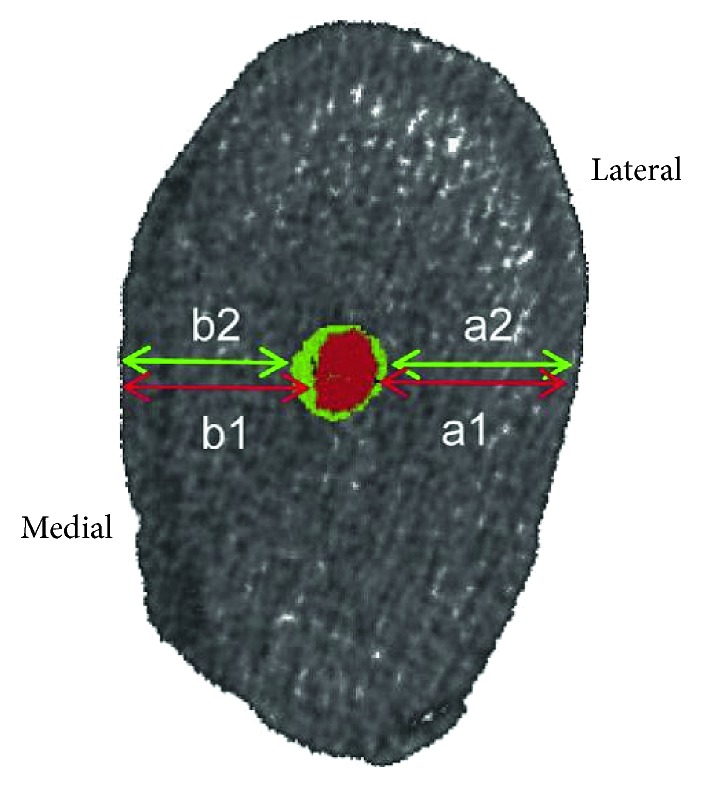
Schematic view of measurement of image cross section. Canal transportation = (*a*1 − *a*2) − (b1 − b2).

The amount of canal transportation was calculated using the following formula: CT = (*a*1 − *a*2) − (*b*1 − *b*2), where *a*1 was the shortest distance from the lateral edge of the uninstrumented canal to the lateral edge of the root, *b*1 was the shortest distance from the medial edge of the uninstrumented canal to the medial edge of the root, *a*2 was the shortest distance from the lateral edge of the instrumented canal to the lateral edge of the root, and *b*2 was the shortest distance from the medial edge of the instrumented canal to the medial edge of the root (Figure 1). Positive values obtained from this formula indicate the occurrence of transportation lateral to the curvature, whereas negative values indicate transportation in a direction facing the furcation.

Based on this formula, zero value indicates no transportation, negative values indicate transportation in the distal direction (furcation side), and positive values indicate transportation in the mesial direction. It should be noted that canal preparation was done by the same operator in all groups, and canal wall thickness was measured by another operator blinded to the group allocation of teeth.

Data were analyzed using SPSS version 20 (SPSS Inc., IL, USA). The Kolmogorov–Smirnov test was used to assess the distribution of data, which showed that data were not normally distributed. Thus, the mean and standard deviation (SD) of root canal transportation were calculated and compared using nonparametric Kruskal–Wallis test. *P* ≤ 0.05 was considered statistically significant.

## 3. Results

**Figure 2:**
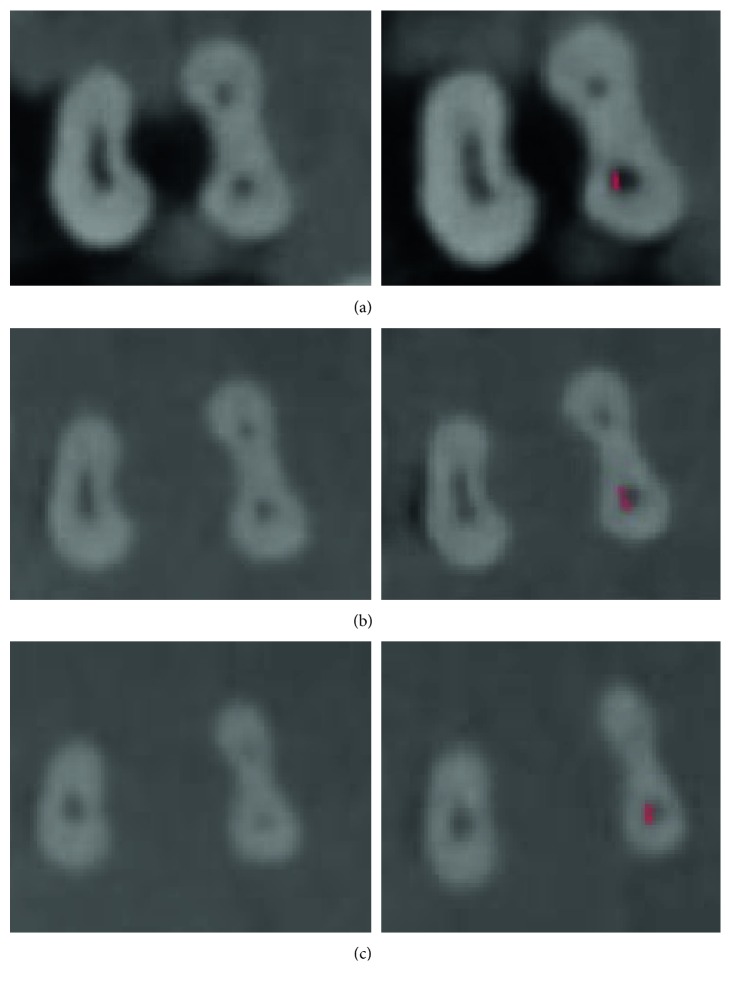
CT scan images before (left column) and after (right column) instrumentation with One Shape. Red marks indicate canal transportation. (a) Coronal third, (b) middle third, and (c) apical third.

Figures 1 and 2 show the schematic view and CT scan images before and after instrumentation in the coronal, middle, and apical cross sections, respectively.

**Figure 3:**
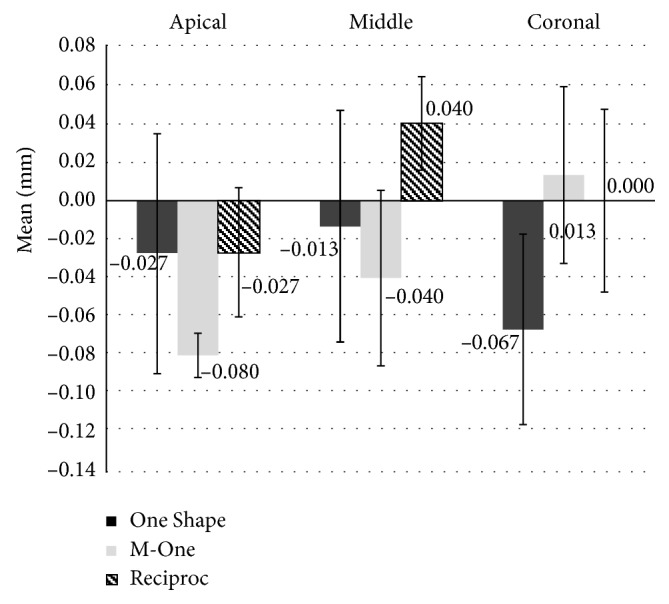
The mean amount of canal transportation in the apical, middle, and coronal thirds in the three groups.

Table 1 and Figure 3 show the amount of canal transportation in the apical, middle, and coronal thirds in the three groups. No significant difference was noted in the amount of canal transportation among the three groups (*P* > 0.05). M-One caused greater transportation in the apical third compared with Reciproc and One Shape, and One Shape caused greater transportation in the coronal third compared with the other groups, although its magnitude was less than 0.3 mm.

## 4. Discussion

The present study compared the amount of canal transportation caused by three single-file systems in root canals using CBCT. Reciproc, One Shape, and M-One were not significantly different in terms of canal transportation. It appears that reciprocating motion causes less transportation compared with full rotation.

Mandibular molars are among the most common teeth requiring endodontic treatment [16, 17]. Thus, quality of root canal preparation in these teeth is an interesting topic of research. Mesial canals of these teeth often have mesiodistal and/or buccolingual curvatures. Due to more severe curves in the mesiobuccal canal, this canal is highly susceptible to transportation during mechanical preparation by endodontic instruments. Canal transportation refers to complete removal of dentin from the external wall of the curvature in the apical half of the canal, which is due to the tendency of file to straighten up and return to its original straight shape during preparation of curved root canals; this may lead to ledge formation and possible perforation of canal. In addition, canal transportation in the coronal third may lead to strip perforation and reduction in residual dentin thickness [18].

Wu et al. [19] reported that apical transportation more than 0.3 mm negatively affects the sealing ability of root filling materials. In our study, canal transportation over 0.3 mm was not seen in any group, and the magnitude of canal transportation was between 0 and 0.08 mm. Our results showed no significant difference in magnitude of canal transportation among the three rotary systems tested. M-One caused greater transportation in the apical third compared with Reciproc and One Shape, and One Shape caused greater transportation in the coronal third compared with other groups, although its magnitude was less than 0.3 mm. However, One Shape showed maximum transportation in the internal wall of the curvature in the coronal third, which can weaken the canal wall and increase the risk of strip perforation and microcrack formation.

Similar to our study, Dhingra et al. [8] showed that One Shape removed more dentin from the coronal third than Reciproc and WaveOne, which may be related to decreased torsional and flexural stresses in reciprocating motion, resulting in higher centering ability and less taper lock [20]. Another study showed that WaveOne with reciprocating motion caused less transportation than One Shape and ProTaper [12]. It appears that One Shape and M-One have higher tendency to remove dentin from the internal wall of the curvature while Reciproc operates in a safer way. This finding can be explained by the difference between the reciprocating and continuous rotational motions.

The reciprocating motion consists of a clockwise motion and a counterclockwise motion and allows the file to be continuously free against the internal wall of the curvature; thus, it operates opposite to the balanced force preparation technique and maintains the central canal path while shaping it. Also, this file is made of M-Wire alloy and has a variable angle and helical pitch, which increase its flexibility. Another study also showed that reciprocating motion, in contrast to continuous rotation, did not increase apical transportation [21].

Saber et al. [13] showed that Reciproc and WaveOne instruments caused significantly less transportation than One Shape, which may be attributed to the use of M-Wire alloy in fabrication of Reciproc and WaveOne files and their reciprocating motion. However, Cimilli and Kartal [14] indicated that continuous rotational motion has higher centering ability compared with reciprocating motion. Moreover, Beurklein et al. [22] indicated that One Shape had higher canal centering ability than Reciproc.

Attempts are ongoing to improve the efficacy of chemomechanical preparation of root canals by new instruments and disinfecting agents. It appears that by use of files with reciprocating motion, compared to those with full rotational movement, optimal shaping of root canals with minimal canal transportation can be achieved.

## 5. Conclusion

The magnitude of canal transportation was not significantly different among different rotary systems in root canal preparation, except for the mean transportation in coronal sections created by M-One rotary file, which was significantly greater than that in the control group.

## Figures and Tables

**Table 1:** The mean amount of canal transportation in the apical, middle, and coronal thirds in the three groups.

Region	Group	Mean amount of transportation (SD)	*P* value
Apical	Reciproc	−0.027 (0.033)	0.103
	One Shape	−0.027 (0.062)	
	M-One	−0.080 (0.011)	

Middle	Reciproc	0.040 (0.024)	0.187
	One Shape	−0.013 (0.060)	
	M-One	−0.040 (0.045)	

Coronal	Reciproc	0.000 (0.047)	0.326
	One Shape	−0.067 (0.049)	
	M-One	0.013 (0.046)	

*P* value: Kruskal–Wallis test.
